# Epstein-Barr Virus-Encoded BILF1 Orthologues From Porcine Lymphotropic Herpesviruses Display Common Molecular Functionality

**DOI:** 10.3389/fendo.2022.862940

**Published:** 2022-05-26

**Authors:** Maša Mavri, Valentina Kubale, Daniel P. Depledge, Jianmin Zuo, Christene A. Huang, Judith Breuer, Milka Vrecl, Michael A. Jarvis, Eva Jarc Jovičić, Toni Petan, Bernhard Ehlers, Mette M. Rosenkilde, Katja Spiess

**Affiliations:** ^1^ Institute of Preclinical Sciences, Veterinary Faculty, University of Ljubljana, Ljubljana, Slovenia; ^2^ Department of Biomedical Sciences, Faculty of Health and Medical Sciences, University of Copenhagen, Copenhagen, Denmark; ^3^ Department of Medicine, New York University School of Medicine, New York, NY, United States; ^4^ Institute of Immunology and Immunotherapy, College of Medical and Dental Sciences, University of Birmingham, Birmingham, United Kingdom; ^5^ Department of Surgery, Division of Plastic & Reconstructive Surgery, Division of Transplant Surgery, Anschutz Medical Campus, University of Colorado, Denver, CO, United States; ^6^ Division of Infection and Immunity, University College London, London, United Kingdom; ^7^ The Vaccine Group Ltd, Plymouth; and the University of Plymouth, Plymouth, United Kingdom; ^8^ Department of Molecular and Biomedical Sciences, Jožef Stefan Institute, Ljubljana, Slovenia; ^9^ Division 12, Measles, Mumps, Rubella, and Viruses Affecting Immunocompromised Patients, Robert Koch Institute, Berlin, Germany

**Keywords:** Epstein-Barr virus, porcine lymphotropic herpesviruses (PLHV), BILF1, G protein signaling, MHC class I, drug target, post-transplant lymphoproliferative disease, in-vivo model

## Abstract

Infection of immunosuppressed transplant patients with the human γ-herpesvirus Epstein-Barr virus (EBV) is associated with post-transplant lymphoproliferative disease (PTLD), an often fatal complication. Immunosuppressed miniature pigs infected with γ-herpesvirus porcine lymphotropic herpesvirus 1 (PLHV1) develop a similar disease, identifying pigs as a potential preclinical model for PTLD in humans. BILF1 is a G protein-coupled receptor (GPCR) encoded by EBV with constitutive activity linked to tumorigenesis and immunoevasive function downregulating MHC-I. In the present study, we compared BILF1-orthologues encoded by the three known PLHVs (PLHV1-3) with EBV-BILF1 to determine pharmacological suitability of BILF1 orthologues as model system to study EBV-BILF1 druggability. Cell surface localization, constitutive internalization, and MHC-I downregulation as well as membrane proximal constitutive Gα_i_ signaling patterns were conserved across all BILFs. Only subtle differences between the individual BILFs were observed in downstream transcription factor activation. Using Illumina sequencing, PLHV1 was observed in lymphatic tissue from PTLD-diseased, but not non-diseased pigs. Importantly, these tissues showed enhanced expression of PLHV1-BILF1 supporting its involvement in PTLD infection.

## 1 Introduction

Epstein-Barr virus (EBV), also known as human herpesvirus 4 (HHV4), belongs to the γ-herpesvirus genus *lymphocryptovirus* and is present in approximately 95% of the adult population worldwide (1). EBV is an oncovirus capable of establishing a lifelong latent infection in memory B cells following primary infection. Although immunocompetent adults usually carry the virus asymptomatically, the yearly global burden of EBV-associated malignancies amounts to approximately 50,000 cases, with nasopharyngeal carcinoma, classical Hodgkin’s lymphoma, Burkitt’s lymphoma and gastric cancer being the most common (2, 3). EBV infection is also a driving factor in the development of post-transplant lymphoproliferative disease (PTLD), a major complication in immunocompromised solid organ (SOT) and hematopoietic stem cell transplantation (HSCT) patients, resulting in tumour development with a high risk of fatal outcome (4–6).

Despite the high incidence of EBV infection within the human population and its direct link to clinical disease, no vaccine or antiviral drug exists to control EBV infection or EBV-associated disease (1, 7). A number of promising antiviral agents that effectively inhibit EBV replication *in vitro* have shown only limited success in clinical trials (8–10). This failure in translation is partly due to the lack of ideal preclinical animal models (11) that mimic physiological, immunological and pathological properties of EBV-associated disease in humans. Mouse models are limited by the strict host tropism of EBV for humans, requiring the use of humanized mouse models (12, 13). Immunodeficient mouse strains (NOG and NSG) reconstituted with human stem cells have been used to study the development of EBV-associated lymphoma or lymphoproliferative disease (12, 14–16) and limited features of primary EBV infection (17, 18). However, major differences in genetics, immunologic and physiologic characteristics between mice and humans complicate direct translation of results from these models into human disease, especially cancer (19).

Non-human primates (NHPs) infected with naturally occurring γ-herpesviruses (*lymphocryptoviruses*) homologous to EBV provide alternative, and arguably more biologically relevant models to study EBV infection and disease. Infection of NHPs with these viruses mimic EBV primary infection in humans (20) as well as PTLD, following experimental immunosuppression and transplantation, where cells in tumour tissue showed evidence of infection (21–24). However, high cost and ethical concerns limit the wide-spread use of these NHP models. Miniature pigs represent an alternative large animal model for PTLD, with the disease developing after experimental immunosuppression during SOT or HSCT (25, 26). PTLD development in these animals is associated with either primary infection or reactivation of porcine lymphotropic herpesvirus 1 (PLHV1), a porcine γ-herpesvirus related to EBV (26–28). Together with two others closely related porcine *macaviruses*, PLHV2 and 3, these three viruses are widespread in pig population and are often co-infecting the host. Moreover, they are all related to EBV in terms of B cell tropism, and sequence similarity of conserved genes (29–31). The association of PTLD with PLHV1, shown by progressive increase of PLHV1 viral load in the diseased animals, has identified this preclinical model as suitable to study pathological aspects of EBV-mediated lymphoproliferative disease (25, 26, 32).

G protein-coupled receptors (GPCRs) serve as drug targets for the treatment of a variety of diseases, with approximately 40% of all approved drugs targeting these molecules (33). Intriguingly, several herpesviruses contain open reading frames (ORFs) that encode viral GPCRs (vGPCRs), which are believed to have been acquired through ancient acts of molecular piracy from the host (34). Examples of vGPCRs include: US28 encoded by human cytomegalovirus (HCMV) (35, 36); ORF74 encoded by human Kaposi’s sarcoma associated herpesvirus (KSHV) (37, 38) and the related equine herpesvirus (39); and BILF1 encoded by EBV and other primate as well as ungulate γ-herpesviruses (40–42), including PLHV1-3 of pigs (29, 43).

BILF1 encoded by EBV (EBV-BILF1) is the most extensively characterized BILF1 receptor. It is an orphan vGPCR with immunoevasive properties associated with MHC-I cell surface downregulation, thereby preventing recognition of EBV-infected cells by CD8+ T cells (44–46). MHC-I downregulation involves interference of EBV-BILF1 with MHC-I endocytic and exocytotic pathways, leading to enhanced lysosomal degradation and reduced presentation of newly synthesized MHC-I molecules at the cell surface (45). EBV-BILF1 is primarily expressed as a late lytic cycle protein, with increasing immunosuppressive activity being observed through progression of the lytic cycle (47). Moreover, EBV-BILF1 has also been detected during latency in clinical samples from Burkitt’s lymphoma patients (48). In addition to immunoevasive properties, EBV-BILF1 acts as an oncogene, inducing tumorigenesis through constitutive activation of Gα_i_-dependent signaling both *in vitro* and *in vivo* (41, 49, 50). Further downstream, EBV-BILF1 induced signaling results in constitutive activation of nuclear factor κ-B (NF-κB) and nuclear factor of activated T cells (NFAT) transcription factors, and inhibition of forskolin-induced transcription of cyclic AMP-responsive elements (CRE) (41, 50, 51). In COS-7 cells and Burkitt’s lymphoma B cells, EBV-BILF1 also downregulates phosphorylation of the double-stranded RNA-dependent protein kinase (PKR) (50).

vGPCRs have been identified as suitable for pharmacological intervention against herpesvirus-infected cells (52). Recently, the structure of EBV-BILF1 was solved using cryo-EM, and revealed substantial differences from closest endogenous GPCR (53), elucidating structural challenges for drug targeting of BILF1 by small molecule inhibitors. A previously published pig model infected with PLHV1 may therefore be a useful preclinical model not only to study EBV associated PTLD disease, but also to test the utility of pharmacological interventions targeting BILF1 as a potential immunotoxin drug target. Towards these aims, additional studies comparing pharmacological properties of the different BILF1 orthologues are warranted.

In the present study, we focused on characterization of BILF1 orthologues from PLHV1-3. We show conservation in BILF1 from EBV and PLHV1-3 regarding cell surface localization, as well as constitutive internalization and ability to downregulate MHC-I. Upstream signaling resulting in Gα_i_-mediated constitutive activation was conserved between orthologues, but PLHV1-3 BILF1 differed in downstream signaling and activation of NF-κB and NFAT transcription factors compared to EBV-BILF1. Finally, we show that only PLHV1, but not PLHV2 or 3, was found in lymphatic tissue from diseased miniature pigs with PTLD, and in these tissues PLHV1-BILF1 was upregulated, which was not observed in non-diseased pigs. Together, these results provide a first step towards establishing a PLHV1-associated PTLD pig model not only for the study of pathological aspects of EBV-mediated disease, but also to test BILF1 as a potential drug target with relevance to treatment of EBV-associated PTLD in humans.

## 2 Materials and Methods

### 2.1 Constructs and Cloning

EBV-BILF1 and the PLHV1-3 BILF1 receptors with an N-terminal FLAG-tag were cloned into the pcDNA3.1+ vector. Gα_Δ6qi4myr_ recombinant G protein was kindly provided by Evi Kostenis (Institute for Pharmaceutical Biology, University of Bonn, Germany).

### 2.2 Cell Culture and Transfection

Human embryonic kidney 293 (HEK-293) cells were cultured at 37°C and 10% CO_2_ in Dulbecco’s modified Eagle’s medium (DMEM; Invitrogen) containing 10% fetal bovine serum (FBS) and 1% penicillin-streptomycin. Porcine kidney 15 (PK-15) cells were cultured at 37°C and 5% CO_2_ in minimum essential medium (MEM; Invitrogen) containing 10% heat inactivated FBS and 1% penicillin-streptomycin. CRISPR/Cas9 modified HEK-293A pan knock-out (KO) cells (ΔGs/olf/q/11/12/13/z) (54) and HEK-293A parental cells were kindly provided by Asuka Inoue (Tohoku University, Japan). They were cultured at 37°C and 5% CO_2_ in DMEM supplemented with 10% heat inactivated FBS, 1% penicillin-streptomycin and 1% L-glutamine. Cells were transfected using Lipofectamine 2000 (Invitrogen) according to the manufacturer’s recommendation. For immunohistochemistry, cells were transfected using Fugene 6 (HEK-293) or FuGeneHD (PK-15) transfection reagent (Promega) according to the manuacturer’s instructions.

### 2.3 Sample Material From PTLD Diseased Pigs

Pig tissue samples were obtained from earlier published *in vivo* experiments that have been described in detail (26, 32, 55, 56). These animals were derived from partially inbred MHC-defined miniature swine herd (Massachusetts General Hospital) and all received 1000cGy thymic irradiation (TI) and 0.05mg/kg pCD3-CRM9 two days prior to cytokine mobilized peripheral blood cell transplantation (57). Oral cyclosporine (Neoral) was started at a dose of 15mg/kg one day before transplantation and was continued BID for 30-60 days (or until death). Samples from three pigs were collected at the time of the transplantation and after the onset of PTLD disease, whereas samples from two PTLD diseased pigs were collected only after disease onset. Samples from one pig without signs of disease were used as controls.

### 2.4 Cell-Based Enzyme-Linked Immunosorbent Assay 

HEK-293 and PK-15 cells were seeded in Poly-D-lysine pre-coated 96-well plates and after 24 hours transiently transfected with various concentrations of receptor DNA (0, 1, 2, 5, 10, 25, 35, 50ng) using Lipofectamine 2000. In order to achieve comparable transfection efficiency in both cell types, we increased the amount of DNA in PK-15 cells. Twenty-four hours after transfection, cells were fixed with 3.7% paraformaldehyde in PBS/CaCl_2_ (pH 7.3) for 10 minutes. After three washes in PBS, cells were blocked with 2% bovine serum albumin (BSA) in PBS/CaCl_2_ for 30 minutes at RT and subsequently incubated with primary mouse M1 anti-FLAG antibody (Sigma-Aldrich) at 1:2250 in 1% BSA/PBS/CaCl_2_ for 1 hour at RT. Following three washes in PBS/CaCl_2_, cells were incubated with secondary goat anti-mouse horseradish peroxidase-conjugated IgG antibody (Sigma-Aldrich) at 1:1000 in PBS/CaCl_2_ for 1 hour at RT. Peroxidase activity was determined by addition of 3,3’-5,5’-tetramethyl benzidine substrate (TMB) (Sigma-Aldrich) for 5 minutes. The reaction was terminated by addition of 0.2M H_2_SO_4_ and absorbance was measured at 450 nm using FlexStation3^®^ Benchtop Multi-Mode Microplate Reader (Molecular devices).

### 2.5 Confocal Microscopy

HEK-293 and PK-15 cells were seeded on fibronectin coated (10µg/mL) coverslips in 24-well plates. The next day cells were transiently transfected using FuGene (HEK-293) or FugeneHD (PK-15). Twenty-four hours after transfection, cells were washed with PBS and fixed in 3.7% paraformaldehyde for 10 minutes on ice and additional 10 minutes at RT. Following three washing steps, cells were permeabilized using 0.02% saponin in 1% donkey serum/PBS and additionally blocked with 10% donkey serum in PBS for 20 minutes. For cell surface receptor visualization, cells were incubated with primary mouse M1 anti-FLAG antibody (Sigma-Aldrich) at 1:2250 in 1% donkey serum/PBS/CaCl_2_ for 1 hour at RT. Following three washes with PBS/CaCl_2_, cells were incubated with secondary donkey anti-mouse Alexa Fluor 594 antibody at 1:100 in PBS for 1 hour at RT. For the last 10 minutes of the incubation, wheat germ agglutinin (WGA) conjugated to Alexa488 (Invitrogen) was added to the cells as a membrane marker (5μg/mL). Cells were additionally incubated with PBS containing Hoechst 33342 stain (Invitrogen) (1μg/mL) and samples were mounted with 8μL of Fluorescence mounting medium (Dako) before imaging on a fluorescence microscope (LSM700).

### 2.6 Luciferase-Based Transcriptional Assay

Luciferase assays were performed on HEK-293 cells or CRISPR/Cas9 genetically engineered HEK-293A cells depleted of different G proteins and parental HEK-293A cells. Cells were seeded at 35,000 cells per well on white 96-well plates pre-treated with poly-D-lysine. The following day, cells were transiently transfected with receptor constructs at indicated concentrations and co-transfected with transcription reporter plasmids CRE-Luc, NFAT-Luc or NF-κB-Luc at 30ng/well using Lipofectamine 2000 (Invitrogen). Additional co-transfection with Gα_Δ6qi4myr_, which is recognized by GPCRs as a Gα_i_ protein but elicits Gα_q_-dependent signaling (58) was performed in CRE and co-transfection with Gα_q_ or Gα_11_ was performed in NFAT luciferase assay. After 24 hours, cells were washed with PBS and incubated for 30 minutes with a mixture of SteadyLite (50μL/well, PerkinElmer) and PBS (50μL/well). Plates were read on an EnVision Multilabel Plate Reader (PerkinElmer) using the luciferase program. The experiment was performed three times in triplicates.

### 2.7 Antibody Feeding Internalization Assay

To determine the internalization properties of BILF1 orthologues, we performed two separate assays, both relying on the principal of antibody uptake over time. With time-course cell-based ELISA assay, we quantitatively assessed the amount of surface expressed receptors over time. Using microscopy approach, surface expressed and internalized receptors, can be visualized in different time points after induction of internalization.

#### 2.7.1 Time-Course Cell-Based ELISA Internalization Assay

Transiently transfected HEK-293 cells were seeded in 24-well plates coated with poly-D-lysine at a density of 2x10^5^ cells/well. After 24 hours, cells were incubated with cold DMEM containing primary mouse M1 anti-FLAG antibody at 2μg/mL (Sigma-Aldrich) at 4°C for 1 hour. Following three washes in cold DMEM, cells were either immediately fixed (t=0) with 3.7% paraformaldehyde or incubated in pre-warmed DMEM media (37°C) at different time points (t=5, 10, 20, 30, 60 minutes) to induce the internalization and then fixed. Thereafter the procedure followed the standard cell-based ELISA protocol. The experiment was performed in triplicate at least three times.

#### 2.7.2 Microscopy Based Internalization Assay

HEK-293 and PK-15 cells were seeded on fibronectin-coated 12mm round coverslips in 24-well plates and transfected the next day using Fugene (HEK-293) or Fugene HD (PK-15). Internalization was studied at three different time points (t=0, 15 and 30 min) each on a separate coverslip. After 24 hours, cells were incubated for 1 hour with primary mouse M1 anti-FLAG antibody (2µg/mL) directly labelling the FLAG-tag on the C-terminus of BILF1 receptors. Primary antibody was added in saturation to ensure the labelling of all surface expressed BILF1 receptors. All the handling was performed at 4°C to prevent internalization. For t=0, cells were immediately fixed with 3.7% paraformaldehyde for 20 minutes at 4°C. To induce internalization of labelled receptors (t=15, 30) cells were first incubated in pre-warmed DMEM at 37°C for 15 or 30 minutes and then fixed. Following three washes, cells were blocked in 2% BSA in PBS for 20 minutes at RT. In the next step, intact cell membrane allowed us to label receptors located at the cell surface of fixed cells with secondary donkey anti-mouse Alexa Fluor 488-conjugated antibody (Thermo Fisher) at 1:500 for 1 hour at RT. After membrane permeabilization step with 0.2% Saponin (Sigma) for 20 minutes, additional incubation with donkey anti-mouse Alexa594 antibody (Sigma-Aldrich) at 1:500 was performed to specifically label the internalized receptors. Following two wash steps, cells were incubated with PBS containing Hoechst 33342 stain (Invitrogen) (1μg/mL). Before imaging on fluorescence microscope (LSM700) cells were mounted with 8μL of Fluorescence mounting medium (Dako).

### 2.8 Flow Cytometry Analysis

HEK-293 cells were seeded in a 6-well plate and transfected using FuGeneHD reagent. Fourty-eight hours after transfection, 5x10^5^ cells/sample were transferred to a tube and stained using APC-labelled anti human HLA-A,B,C (W6/32) antibody at the recommended concentration (Biolegend). Receptors were detected using anti-FLAG FITC conjugated antibodies (Genscript) and isotype controls were used to differentiate non-specific background signal from specific antibody signals. Samples were analysed on a BD FACSCanto™ II instrument equipped with 488 nm and 633 nm lasers using FITC (530/30) and APC (660/20) filters. Data were analysed using Kaluza software.

### 2.9 Microscopy-Based Approach Observing MHC-I Downregulation

For analysis of MHC-I downregulation, cells were seeded, transfected and fixed as described above (paragraph 2.5). Afterwards, cells were incubated with primary rabbit anti-FLAG antibody (Sigma-Aldrich) at 1:300 and mouse anti human HLA-A, B, C class I antibody (1:100) in HEK-293 cells (Santa Cruz) or mouse anti-pig SLA class I antibody (1:100) for PK-15 cells (R&D systems) for 1 hour at RT. Following three washes with PBS/CaCl_2_, cells were incubated with secondary goat anti-rabbit Alexa Fluor 647 antibody and goat anti-mouse Alexa Fluor 555 antibody at 1:500 in PBS for 1 hour at RT. For the last 10 minutes of the incubation, WGA conjugated to Alexa488 (Invitrogen) was added to the cells as a membrane marker (5μg/mL). Following two wash steps, cells were incubated with PBS containing Hoechst 33342 stain (Invitrogen) (1μg/mL). Before imaging on a fluorescence microscope (LSM700) cells were mounted with 8μL of Fluorescence mounting medium (Dako). Equivalent numbers of transfected and non-transfected cells were blindly selected and we measured the intensity of 555 channel (MHC-I) using FIJI software.

### 2.10 CellTiter Glo^®^ Viability Assay

Cell viability assay was performed as described in the manufacturer’s manual. Briefly, CRISPR/Cas9 genetically engineered HEK-293A cells depleted of different G proteins and parental HEK-293A cells were seeded at 35,000 cells per well and transfected the next day. 24 hours after transfection, plates were incubated at RT for approximately 30 minutes. 100 µL of CellTiter Glo reagent was carefully added to each well. Plates were incubated for 12 minutes on shaker before the luminescence was measured on EnVision Multilabel Plate Reader (PerkinElmer).

### 2.11 Sequencing of PLHV Infected Pigs

Sequencing libraries were prepared from DNA extracted from four PTLD diseased pig samples using the NEBNext Ultra II DNA Library Prep Kit for Illumina (New England Biolabs), according to manufacturer’s instructions. Libraries were subsequently multiplexed and sequenced using a 300 cycle mid-output kit (2x150 paired-end mode) on a NextSeq 550 (Illumina Technologies).

### 2.12 Competitive Alignment

Prior to competitive alignment, sequence read datasets were quality and adapter trimmed using TrimGalore (https://www.bioinformatics.babraham.ac.uk/projects/trim_galore/). For the competitive alignment step, a hybrid reference genome was constructed by merging FASTA files containing the Sus scrofa (Sscrofa11.1) genome and partial genome sequences for PLHV1 (NC_038264.1), PLHV2 (NC_038265.1), and PLHV3 (AY170316). Sequence read datasets were aligned against the hybrid genome using bbmap (https://sourceforge.net/projects/bbmap/) with minid=0.9 and ambiguous=random before post-processing with SAMtools (59) and BEDtools (60). Coverage plots were generated using the R 4.0 bioconductor packages Gviz (61) and GenomicFeatures (62).

### 2.13 Real-Time qPCR

RNA was extracted from samples using QIAzol^®^ lysis reagent (Qiagen). RNA (500ng) was used to prepare cDNA using Superscript III reverse transcriptase (Thermo Fischer Scientific). RT-qPCR was performed on 96-well plates using TaqMan^®^ Fast Advanced Master mix (2x) (Thermo Fischer Scientific) and costumed TaqMan^®^ gene expression assay probes (Thermo Fischer Scientific), designed for specific detection of PLHV1-BILF1 (forward primer: 5’TCTGATACTCACTGTTGCTAACTTTGTT3’, reverse primer: 5’ACTTATAGCTTGGCGACACTTGAA3’, probe: 5’FAM-ACCTTCAAAGCTCAAAGTAGT-MGB3’), PLHV2-BILF1 (forward primer: 5’GCTGTTGCTAACTTTCTTAvGTTTTGGA3’, reverse primer: 5’ACTTATACCTTGGCGACACTTGAAG3’, probe: 5’FAM-CCTTCAAAGCTCAATACAGC-MGB3’), PLHV3-BILF1 (forward primer: 5’GTCTTTTGGCAGTTGTTGCTAATCT3’, reverse primer: 5’ACTTGTAGCCTGGCGACATT3’, probe: 5’FAM-CAAGTGCAGCATAATTC-MGB3’) and a reference gene RPL4 (assay identification number: Ss03374063_g1). RT-qPCR was performed on a QuantStudio 6 Flex Real-Time PCR system (Thermo Fisher Scientific) using a standard protocol. Ct (cycle threshold) values were acquired using QuantStudio Real-Time PCR software. To present the relative fold change of cDNA levels compared to a reference gene (RPL4) we used 2^ΔCt^ calculation.

### 2.14 Homology Modelling of MHC Class I Molecules

Compared PDB entries: 3BO8 (human) (https://www.rcsb.org/structure/3bo8) & 5NPZ (porcine) (https://www.rcsb.org/structure/5NPZ).

### 2.15 Data Analysis and Statistics

Data were analysed using Graph Pad Prism (8.3.0), Kaluza analysis software and FIJI software and reported as mean ± SEM (standard error of the mean). For all microscopy experiments, images were visualized using an LSM700 microscope and ZEN blue software. Statistical analysis was performed with Graph Pad Prism using ANOVA or student t-test as indicated. P value < 0.05 was considered statistically significant.

## 3 Results

### 3.1 PLHV- and EBV-BILF1 Orthologues Exhibit a Similar Subcellular Localization

**Figure 1:**
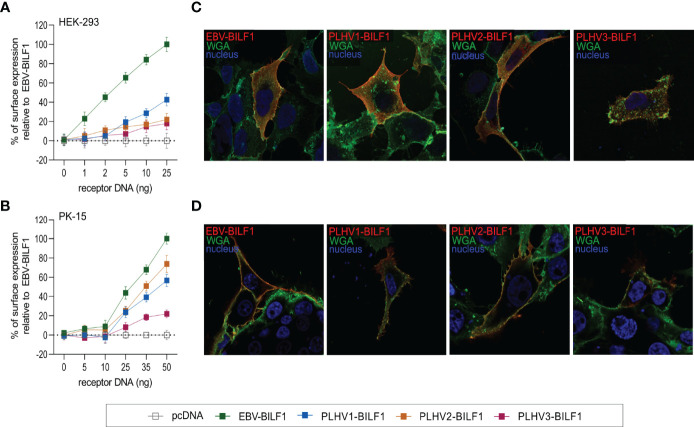
Cell surface expression of BILF1 receptors in HEK-293 and PK-15 cells. **(A, B)** Graphs show surface expression of PLHV BILF1 orthologues compared to EBV-BILF1 expression using cell-based ELISA at increasing concentrations of receptor DNA in **(A)** HEK-293 and **(B)** PK-15 cells (values are mean ± SEM; *n* = 3). **(C, D)** Representative microscopy images show the localization of BILF1 orthologues (red) at the cell surface determined by co-localization with WGA conjugated to Alexa 488 (green) in **(C)** HEK-293 and **(D)** PK-15 cells. Nuclei are stained with Hoechst 33342 stain (blue). Images were taken with 63 × oil immersion plan‐apochromat objective.

EBV-BILF1 is known to localize primarily to the cell surface, which is based on results from a number of studies performed in a variety of cell types [e.g. human embryonic kidney (HEK-293) cells and human melanoma (MJS) cells] (41, 44, 46, 49, 51). To examine the subcellular localization of the three uncharacterized PLHV1-3 BILFs in comparison to EBV-BILF1 in the present study, we cloned BILFs into eukaryotic expression plasmids followed by transient expression in HEK-293 and porcine kidney epithelial (PK-15) cells. Localization was then analysed using both a quantitative cell-based ELISA and fluorescence microscopy. The cell-based ELISA using increasing concentrations of EBV-BILF1 and PLHV1-3 BILF1 expression plasmids, showed cell surface localization of all BILFs in both HEK-293 (**Figure 1A**) and PK-15 cells (**Figure 1B**). To achieve a comparable dose response in both cell lines, higher amounts of plasmid DNA for each BILF1 orthologue was required for transfection in PK-15 cells (**Supplementary Figure 1**). PLHV1-3 BILF1 showed ~20-40% of surface expression levels compared to EBV-BILF1 in HEK-293 cells, and ~60-70% (PLHV1- and 2-BILF1) and ~20% (PLHV3-BILF1) of EBV-BILF1 levels in PK-15 cells. Subcellular distribution determined by fluorescence microscopy using WGA as a cell surface (plasma membrane) marker was consistent with the cell-based ELISA results, as BILFs co-localized with WGA at the cell surface of HEK-293 (**Figure 1C**) and PK-15 cells (**Figure 1D**). In summary, analysis of subcellular distribution shows that similar to EBV-BILF1, PLHV1-3 BILFs are localized at the plasma membrane, and this appears to be independent of cell type.

### 3.2 Gα_i_-Dependent Constitutive Signaling is Conserved Across BILF1 Receptor Orthologues From PLHV1-3 and EBV

**Figure 2:**
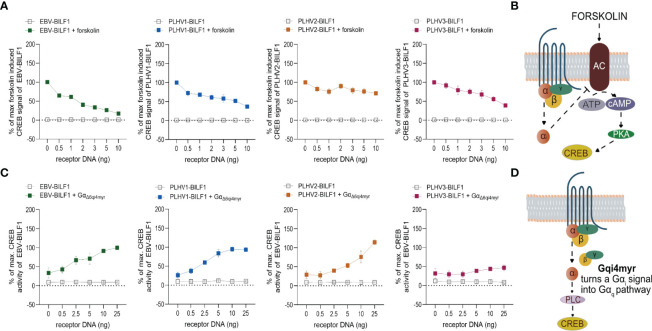
BILF1 receptor signaling is Gα_i_ dependent. **(A)** HEK-293A or **(C)** HEK-293 cells were transiently co-transfected with different concentrations of BILF1 orthologues 30ng/well of CRE cis-reporter plasmid. **(A)** HEK-293A cells were stimulated with 10µM of forskolin for 5 hours, before gene-dose dependant CRE activity of BILF1 receptors was measured. **(C)** HEK-293 cells were additionally co-transfected with 30ng/well of Gα_Δ6qi4myr_ and CRE activity was measured 24 hours after transfection (values are means ± SEM; *n* = 3). **(B)** Forskolin induces cyclic AMP formation and enables the observation of its decrease mediated by receptors. **(D)** Chimeric G protein Gα_Δ6qi4myr_ is recognized by receptor as a Gα_i_, but functions as a Gα_q_ subunit, resulting in increased activity of phospholipase C (PLC) and hence CRE activity.

Next, we focused on the membrane proximal signaling pattern of PLHV1-3 BILFs in comparison to the previously described Gα_i_-dependent signaling of EBV-BILF1 (41, 50). For these studies we used HEK-293 cells, which is a cell-type commonly used for studying signaling properties of GPCRs (63) also based on their expression of full repertoire of G protein subunits (64). We first investigated receptor-mediated regulation on the activity of the downstream cyclic adenosine monophosphate (cAMP) response element (CRE) transcription factor (**Figure 2**), which responds to receptor activity *via* Gα_s_ (increased activity) and Gα_i_ (decreased activity) (65). Cells were pre-treated with forskolin, an inducer of cAMP formation, which enables measurement of Gα_i_-coupled receptor activity (**Figures 2A, B**). Similar to EBV-BILF1, PLHV1-3 BILFs inhibited forskolin-induced CRE transcription factor activity in a dose dependent manner (**Figure 2A**). Among PLHV BILFs, PLHV2-BILF1 showed a moderate (30%) decrease in forskolin induced CRE transcription factor activity in comparison with BILF1 from PLHV1 and PLHV3, where the level of inhibition was approximately 2-fold higher (approx. 60% decrease) and comparable to EBV-BILF1 levels. In the absence of forskolin, receptor-mediated CRE transcription factor activity was not detected for any BILF1 orthologue, excluding Gα_s_ dependent activation. To support these findings, we co-transfected HEK-293 cells with the different BILFs together with the chimeric G protein Gα_Δ6qi4myr_ (**Figures 2C, D**). Activation of this chimeric protein leads to increased CRE activity through phospholipase C (PLC). Using this approach, BILF1 from both PLHV1 and PLHV2 increased CRE activity in a gene dose-dependent manner to a similar level observed for EBV-BILF1; PLHV3-BILF1 showed lower (~46%) activity. Together, our analysis showing inhibition of both forskolin-dependent CRE transcription factor activity and increased CRE activity following co-transfection with Gα_Δ6qi4myr_ indicate that constitutive membrane proximal signaling *via* Gα_i_ is generally conserved across PLHV1-3 BILFs similar to EBV-BILF1 (41, 50).

### 3.3 EBV and PLHV1-3 BILF1 Receptors Show Differential Membrane Distal Signaling in Activation of NFAT and NF-κB Transcription Factors

**Figure 3:**
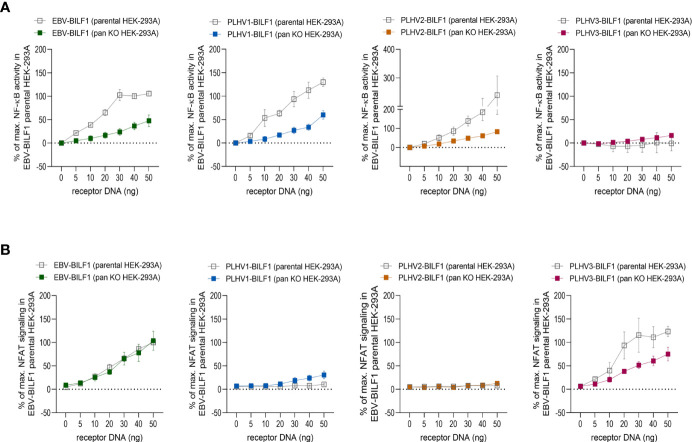
BILF1 receptor signaling in CRISPR/Cas9 modified HEK-293A cells. **(A)** NF-κB and **(B)** NFAT activity was determined in parental HEK-293A cells and HEK-293A cells depleted of various G proteins (ΔGα_s/olf/q/11/12/13/z_) by CRISPR/Cas9 technology (pan KO HEK-293A cells). We used 30ng/well of **(A)** NF-κB and **(B)** NFAT cis-reporter plasmid. Graphs represent the receptor dependent NF-κB and NFAT activity relative to the maximal activity of EBV-BILF1 in parental cells (values are means ± SEM, *n* = 3).

In addition to CRE, EBV-BILF1 activates more distally located downstream NF-κB and NFAT transcription factor pathways (45, 46, 50, 51). To test the capacity of PLHV1-3 BILFs for activation of these additional signaling pathways, we first examined constitutive activation of NF-κB and NFAT in HEK-293A (parental) cells. **Figure 3** and **Supplementary Figure 2** show the activity of PLHV1-3 BILF1 receptors relative to the maximal NF-κB or NFAT activity elicited by EBV-BILF1 in parental cells (designated as 100% activity). EBV-BILF1 activated both NF-κB and NFAT transcription factors. BILF1 from PLHV1 and PLHV2 showed ~130% and ~240% NF-κB activity, respectively (**Figure 3A** and **Supplementary Figure 2A**) compared to EBV-BILF1 and either low or no NFAT activity, respectively. PLHV3-BILF1 failed to activate NF-κB, but showed comparable NFAT activity (~120%) to EBV-BILF1 in these cells (**Figure 3B** and **Supplementary Figure 2B**). In summary, these results from parental cells show that only PLHV1- and PLHV2-BILF1 can mediate NF-κB activity, whereas NFAT activity is only induced by PLHV3-BILF1 in comparison to EBV-BILF1, which is able to activate both transcription factors.

To determine the dependence of the observed NF-κB and NFAT activity on Gα_i_, we then used HEK-293A cells genetically engineered by using CRISPR/Cas9 to express only Gα_i_ proteins (ΔG_s/olf/q/11/12/13/z_; “pan KO” cells). As shown in **Figure 3A**, BILF1 from EBV, PLHV1 and PLHV2 retained NF-κB activity in pan KO cells (50%, 50% and 80%, respectively, compared to EBV-BILF1 activity in parental cells), showing important but not exclusive Gα_i_ mediated NF-κB activation for these receptors. Again, comparable to its behaviour in parental cells, PLHV3-BILF1 did not show any significant activity in pan KO cells (**Figure 3A** and **Supplementary Figure 2A**). NFAT activity of EBV and PLHV3 -BILF1 (**Figure 3B** and **Supplementary Figure 2B**) was conserved in pan KO cells (100% and ~75%, respectively) implicating Gα_i_ as the primary G protein involved in NFAT signaling through these receptors. Importantly, cell surface expression of BILFs was approximately 20% lower in pan KO cells and comparable to our results shown on **Figure 1** with EBV-BILF1 showing highest cell surface expression (**Supplementary Figure 3**). Surprisingly, in pan KO cells PLHV1-BILF1 showed ~30% activity, whereas in parental cells it did not show any activity. Consistent with NFAT signaling in parental cells, PLHV2-BILF1 did not activate NFAT (**Figure 3B** and **Supplementary Figure 2B**). In summary, only PLHV3-BILF1 showed NFAT activity comparable to EBV-BILF1.

Together, results from these studies show that Gα_i_-coupling is conserved between all BILFs at membrane proximal levels, but differs for the activation of more distal levels of NF-κB and NFAT transcription factors.

### 3.4 EBV and PLHV1-3 BILF1 Receptors Undergo Constitutive Internalization

**Figure 4:**
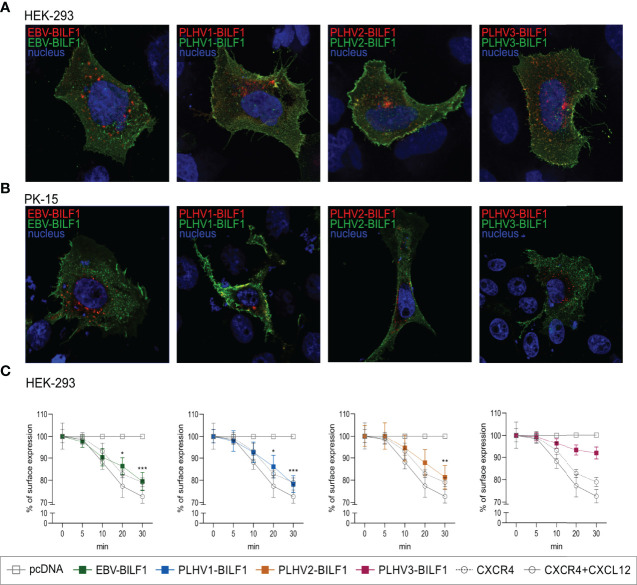
Constitutive internalization of the BILF1 receptor family. **(A, B)** Antibody feeding based microscopy assay showed internalization of BILF1 receptors in **(A)** HEK-293 cells and **(B)** PK-15 cells. Alexa488 antibody (green) was used to label receptors expressed at the cell surface and Alexa 594 antibody (red) to detect the internalized receptors. Nuclei are stained with Hoechst 33342 stain (blue). Images were taken with 63 × oil immersion plan‐apochromat objective. **(C)** Time-course cell-based ELISA internalization assay. Receptor surface expression was determined over time as in cell-based ELISA experiment. CXCR4 was additionally incubated with 10µM CXCL12 as a positive control (values are means ± SEM, *n* = 3). Statistics were performed by 2-way ANOVA using GraphPad Prism. *p–value < 0.05; **p–value < 0.01; ***p–value < 0.001.

Constitutive internalization of EBV-BILF1 has previously been proposed both as a means to function as an immunoevasin downregulating MHC-I molecules, and as an important regulatory mechanism to enable constitutive signaling during the virus replication cycle (44). We next determined the internalization of PLHV1-3 BILFs by using antibody feeding experiments in combination with fluorescence microscopy and cell-based ELISA. Microscopy analysis confirmed that all BILFs were located at the cell surface at the beginning of the experiment (t=0). Additionally, the constitutively internalized fraction of the receptors were shown to localize to a comparable perinuclear endocytic vesicular site after 30 minutes in both HEK-293 and PK-15 cells (**Figures 4A, B**). Time-dependent receptor internalization was analysed by cell-based ELISA in HEK-293 cells where BILF1 surface expression was measured following incubation at 37°C for 0, 5, 10, 20 and 30 minutes (**Figure 4C**). Internalization of PLHV1 and PLHV2 BILF1 receptors showed comparable rates of internalization as EBV-BILF1, with ~20% of these receptors being internalized after 30 minutes. Internalization of PLHV3-BILF1 was slower with only 8% being internalized over the same time period. Thus, in terms of rate and total level of internalization as well as subcellular distribution following internalization, BILFs from PLHV1 and 2 are similar to the constitutively internalizing EBV-BILF1 receptor.

### 3.5 Immunoevasive Properties of BILF1 Receptor Family

**Figure 5:**
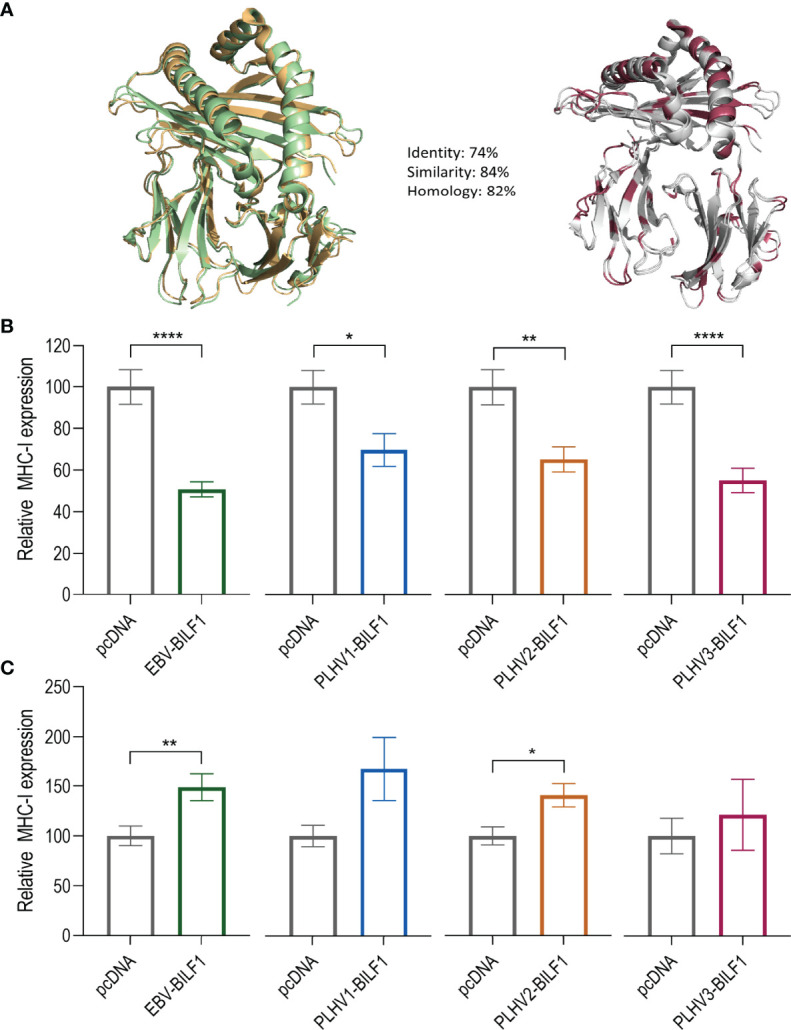
PLHV1-3 BILFs downregulate surface MHC-I molecules. **(A)** Porcine SLA-I molecule (green) compared to the human HLA-I molecule (orange). The differences between the molecules are marked in red. **(B)** HEK-293 and **(C)** PK-15 cells were transfected with FLAG-labelled BILF1 orthologues and stained with rabbit anti-FLAG (receptor labelling) and mouse anti-human HLA class I antibodies. Graphs represent the percentage of surface expressed MHC-I molecules at the surface of 33 BILF1-transfected and 33 non-transfected cells. Statistics were performed by unpaired student t-test using GraphPad Prism. *p–value < 0.05; **p–value < 0.01; ****p–value < 0.0001.

Given the suggested link between constitutive internalization and immunoevasive properties shown for EBV-BILF1 (45, 46) and the observed conserved levels of constitutive internalization for at least two of the PLHV BILF1 receptors, we next determined their immunoevasive properties. Initially, we performed FACS analysis, measuring the expression of endogenous MHC-I molecules at the surface of BILF1 transfected HEK-293 cells in comparison to control (pcDNA) transfected HEK-293 cells as described previously for EBV-BILF1 (44). Our decision to use human HEK293 cells, was supported by comparing the sequence relationship between human and porcine MHC-I with a homology model (**Figure 5A**) showing a 74% aa sequence homology and high structure identity. Our FACS results showed a comparable downregulation of MHC-I in EBV-BILF1 transfected cells as had been previously observed (44) (**Supplementary Figure 4**). Ratio between BILF1 transfected and non-transfected cells showed significantly lower MHC-I expression (0,61 and 0,67) for EBV-BILF1 and PLHV3-BILF1 respectively, whereas PLHV1- and PLHV2-BILF1 showed a tendency towards MHC-I downregulation (0,84 and 0,83 respectively) (**Supplementary Figure 4**). Considering low transfection efficiency observed for all PLHV BILFs which persisted despite optimization using higher concentrations of DNA, different transfection protocols or different vector systems (pcDNA and bicistronic IRES vector) we looked at the single cell MHC-I expression using a new methodology based on fluorescence microscopy (**Figures 5B, C**). This approach allowed us to determine both the receptor expression and to evaluate MHC-I surface expression on transfected (coloured bars) compared to non-transfected (grey bars) cells. Notably, this approach was not limited by transfection efficiency enabling analysis in both human (HEK-293) and porcine (PK15) cells. Although not definitive, the level of homology conservation for human and porcine MHC-I molecules shown (**Figure 5A**), supports the possibility for cross-species MHC-I recognition. As shown in **Figure 5**, all BILF1 receptors significantly reduced surface MHC-I expression in HEK-293 cells. EBV-BILF1 and PLHV3-BILF1 showed approximately 50% downregulation of MHC-I molecules, whereas PLHV1-BILF1 and PLHV2-BILF1 showed 30-35% downregulation of surface MHC-I molecules compared to non-transfected cells (**Figure 5B**). Importantly, FACS data indicated the conserved MHC-I downregulation for PLHV BILF1 receptors, which was further confirmed by microscopy studies in HEK-293 cells. In contrast to HEK-293 cells, in PK-15 cells all BILFs including EBV-BILF1 showed higher expression of MHC-I molecules at the cell surface in transfected cells in comparison to non-transfected cells (**Figure 5C**). This is in contrast to what has been published for EBV-BILF1 and our observations for EBV- and PLHV1-3 BILF1 in HEK-293 cells, therefore we conclude that downregulation of MHC-I cell surface expression mediated by BILF1 is cell type dependent.

### 3.6 PLHV1-BILF1 Expression Levels Are Increased in PTLD Samples

**Figure 6:**
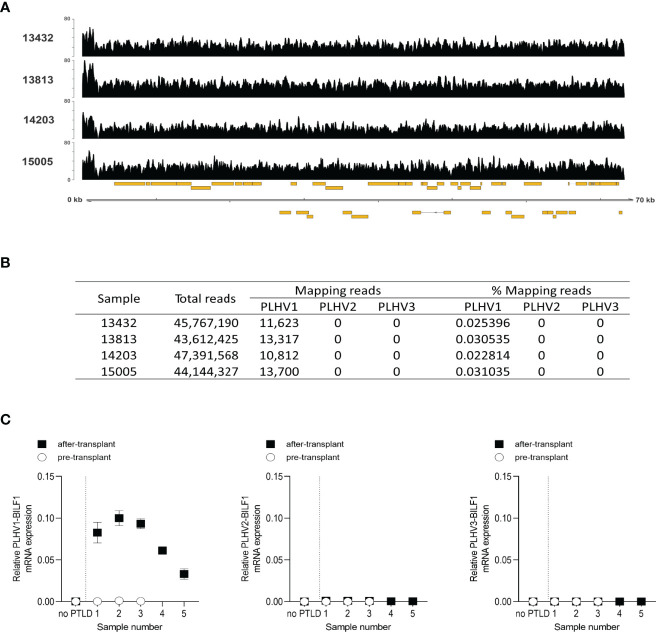
High expression levels of PLHV1 and PLHV1-BILF1 detected in PTLD-diseased pigs. **(A)** Illumina sequencing of PTLD samples allowed the identification of a small number of sequence reads aligning to the PLHV1 UL region. Coverage plots are shown for each sample and show an even distribution across the genomic fragment. The y-axis scale indicates read depth while the x-axis shows the genome fragment along with annotated ORFs (gold boxes). **(B)** Competitive alignment of Illumina sequence reads derived from tumours, against PLHV1, PLHV2, and PLHV3 genome fragments. **(C)** The expression levels of PLHV1-3 BILF1 were determined by qPCR using cDNA from lymphoid tissue samples of PTLD diseased pigs collected before and after disease onset. Results are mean ± SEM of raw data and presented as 2^ΔCt^.

Previously, Huang et al. (2001) reported the development of PTLD in miniature pigs undergoing SOT or HSCT transplantation and immunosuppression, which was associated with PLHV1 infection (26). Notably, histological and pathological properties of PTLD in these pigs resembled the disease in humans, with B-cell lymphoproliferation in peripheral blood, lymph nodes and tonsils. We therefore examined PLHV1-3 expression in samples obtained from four miniature pigs with PTLD by using Illumina sequencing. Further supporting the association of PLHV1 with PTLD, as only PLHV1 was detected in the diseased pigs (**Figures 6A, B**). By contrast, neither PLHV2 nor PLHV3 genomes were detected in any of the sequence data for these samples (**Figure 6B**). Next, to examine the association of PLHV1-BILF1 expression with PTLD in these pigs, we designed specific probes and performed PLHV1-BILF1 qPCR. Notably, in five of five samples collected after the PTLD onset, we detected high expression levels of PLHV1-BILF1 (**Figure 6C**). Importantly, PLHV1-BILF1 was not detected in any of three pre-transplant samples from these same animals that developed disease, nor in a sample from a pig lacking PTLD symptoms, showing specific link between PLHV1 infection and PTLD development. BILF1 from PLHV2 or PLHV3 were not detected in any of the pigs (**Figure 6C**).

## 4 Discussion

In the present study, we compared the localization, signaling, trafficking and immunoevasive properties of PLHV1-3 BILFs with EBV-BILF1. We show that cell surface localization and constitutive internalization are conserved features among all BILFs in both human and porcine cell lines; the ability to downregulate MHC-I molecules from the cell surface was observed only in human HEK-293. Analysis of BILFs signaling pathways showed conserved constitutive signaling mediated predominantly through Gα_i_. However, activation of NFAT and Nf-κB transcription factors differed for PLHV1-3 BILFs compared to EBV-BILF1. Consistent with the association of PLHV-1 with PTLD disease, PLHV-1 infection and increased expression of PLHV1-BILF1 was observed in samples from PTLD diseased pigs.

Constitutive activity is a conserved property among vGPCRs and has been linked to their transforming potential (66, 67). Constitutive signaling and internalization have been reported for HCMV-US28 and KSHV-ORF74 (68, 69) as well as for EBV-BILF1 and BILFs of non-human primate *lymphocryptoviruses* (51). Similar to EBV-BILF1, the recognition of KSHV-ORF74 as oncogene driving several virus associated malignancies was shown to result from the constitutive activation of multiple signaling pathways, but also from ligand dependent activation (e.g., Gα_q_, Gα_i_, Gα_12/13_, MAP kinases and transcription factors NF-κB, NFAT and CRE) (38, 70–73). HCMV-US28 functions as an onco-modulator, however direct oncogenic potential of this receptor remains to be described (74, 75). In the present study, we found that all PLHV1-3 BILFs display constitutive, Gα_i_ dependent signaling. This suggests that like EBV-BILF1, PLHV1-3 BILFs could have cell transforming properties and thereby behave as oncogenes. Previous reports showed constitutive, Gα_i_ mediated activation of NF-κB and NFAT transcription factors for EBV-BILF1 in HEK-293 and COS-7 cells (50, 51). In our study, use of CRISPR/Cas9 modified cells provided a novel system to dissect the involvement of Gα_i_ protein in downstream activation of NF-κB and NFAT transcription factor for EBV and PLHV1-3 encoded BILFs. NF-κB regulates cytokine, chemokine and growth factor secretion (76), whereas NFAT plays an important role in immune system function as well as cell proliferation and apoptosis (77). Activation of both transcription factors has also been confirmed for KSHV-ORF74 and HCMV-US28 where their activation has been linked to regulation of other downstream effectors (COX-2, CXCL8, ANGPT2, ICAM-1) contributing to different cancer hallmarks such as angiogenesis, production of inflammatory cytokines and tumour promoting properties (51, 67, 78). For EBV-BILF1 it was recently shown that NF-κB activation up-regulates intracellular adhesion molecule-1 (ICAM-1), a factor known for its involvement in progression of malignant cancer (79). Our results show that NF-κB activity was conserved for PLHV1- and PLHV2-BILF1 to comparable levels as EBV-BILF1, whereas PLHV3-BILF1 did not induce NF-κB activation. As cell viability was comparable (**Supplementary Figure 5**), the ~50% lower activity in cells expressing only Gα_i_ (pan KO) compared to cells expressing a total repertoire of G proteins (parental) may indicate the involvement of the β/γ subunit or other signaling pathways contributing to the activation of NF-κB (80). Such promiscuous G protein coupling has already been shown for HCMV-US28, where coupling to different G proteins also depends on ligand binding and cell type (81–83). NFAT activation by BILFs is only conserved among BILF1 encoded by EBV, marmoset and siamang herpesvirus (51). In contrast to the activation of NF-κB, we found that NFAT was only activated by PLHV3-BILF1 to a level comparable to that observed for EBV-BILF1, showing some differences in PLHV BILF1 receptor downstream signaling. Importantly, the activity of all PLHV BILF1 orthologues was mediated by Gα_i_ protein, comparable with the observed and previously reported EBV-BILF1 activity.

As mentioned above, constitutive internalization has been proposed as a mechanism required for downregulation of surface MHC-I molecules by EBV-BILF1 (44). Here, we first show conserved constitutive internalization for PLHV1-3 BILFs expressed in HEK-293 and PK-15 cells and further confirm the MHC-I downregulation mediated by these orthologues in HEK-293 cells. Our FACS studies show the conservation of downregulation effect for at least PLHV3-BILF1, where the MHC-I expression at the surface was significantly reduced. However, we believe that low transfection efficiency of PLHV1-3 BILF1 in HEK-293 cells both in terms of percentage of transfected cells and intensity of expression made it more difficult to study these receptors in FACS studies. Therefore, we further applied a single-cell microscopy-based approach, allowing us to study the downregulation in cells with high expression levels of PLHV1-3 BILFs in both HEK-293 and PK-15 cells. When studying BILF1 receptors with low expression profiles (**Supplementary Figure 4**) our novel single-cell microscopy method has the advantage of measuring the fluorescence intensity of the conjugated MHC-I molecule at the cell surface on an individual single cell basis. Importantly, these MHC-I downregulation experiments supported our initial FACS results and suggested a conserved immunoevasive property for all PLHV1-3 BILFs compared to EBV-BILF1 in HEK-293 cells. This method also has some limitations, among which is the small number of cells analyzed. However, we would suggest that it allowed us to reliably investigate whether downregulation is present in cells expressing higher levels of PLHV1-3 BILF1 (**Supplementary Figure 6**). The high sequence identity of human and porcine MHC-I molecules, (**Figure 5A**) may explain why PLHV1-3 BILFs are able to downregulate human MHC-I molecules. However, surprisingly MHC-I downregulation experiments performed in PK-15 cells showed that neither EBV-BILF1 nor any PLHV1-3 BILF was able to downregulate porcine MHC-I molecules in these cells. It is presently unclear as to why downregulation was not observed in porcine cells, especially for PLHV BILFs, but shows that the choice of cell type could be important to study this vGPCR property. B-cells, in which γ-herpesviruses establish a latent infection, may be the most appropriate tissue model to study BILF1 mediated MHC-I downregulation. With PLHV1-3 frequently naturally occurring in pigs, it is very work- and cost intensive to isolate B-cells from seronegative piglets being bottle feed, as negative controls. Moreover, the whole genome sequence of PLHV1-3 is until to date not available, and therefore B-cells cannot be infected with engineered PLHV1-3- or the deletion viruses. These experiments are out of scope of this study but are of high relevance for future studies to establish this potential *in vivo* model.

PTLD disease in pigs, which has been associated with PLHV1 infection, has been shown to model EBV-associated PTLD in humans after HCT and SOT, with disease being a response to either primary infection or reactivation with PLHV1 (26, 27, 32). Similar to PTLD in humans (and NHPs), immunosuppression is an additional requirement for PTLD in PLHV1-infected pigs, supporting similar disease aetiologies. Pigs could therefore serve as an alternative large animal model for PTLD disease. In this study, we confirmed PLHV1 infection in PTLD diseased pigs and enhanced expression of only PLHV1-BILF after disease onset, which further supports the involvement of PLHV1 infection in PTLD. We cannot yet conclude whether enhanced expression of BILF1 encoded by PLHV1 is a result of viral reactivation or upregulation of BILF1. Although presently lacking, annotation of the whole genome sequence of PLHV1 is ongoing and such important questions will be part of future studies being beyond the scope of the present study.

The degree of immunosuppression administered to prevent graft rejection prior to organ transplantation is an important risk factor in EBV-PTLD development (84), as the disease results from uncontrolled expansion of EBV positive B cells from either donor or recipient. Reconstitution of EBV specific cytotoxic T cells in the donor prevents the development of EBV-PTLD, demonstrating that reconstituted immunity against EBV is a successful approach against PTLD (85, 86). As EBV-BILF1 reduces CD8+ T recognition of infected cells by manipulating MHC-I expression, targeting BILF1 to suppress its function may allow better immune recognition and elimination of EBV infected cells by T-cells. At a wider perspective, a reliable *in vivo* model is needed to study EBV associated diseases and potential drug targets against EBV. Similar disease aetiology and the demonstration of high homology between EBV-BILF1 and PLHV1-BILF1 in terms of signaling, internalization and MHC-I downregulation in the present study, further suggests the use of PLHV1-infected pigs as a relevant pre-clinical large animal model for this disease. Human PTLD samples are mostly limited to serum due to the technical challenges of conserving whole blood over time. The large size and long-life span of pigs enables greater ease of sample collection and monitoring over extended time periods (19, 25, 27, 87, 88), where the expression of BILF1 receptor can be determined from isolated B cells, as well as tissue material, such as spleen and lymph nodes, during PTLD development. Such a model would also enable use of genetically modified PLHV1-3 BILFs to study the importance of individual viral genes in PTLD development.

Constitutive receptor internalization is a requirement for drug targeting, as shown for HCMV-US28. Taking advantage of its constitutive internalization and selective binding profile for the chemokine CX_3_CL1, HCMV-US28 has been successfully targeted by a CX_3_CL1-based immunotoxin (89–92). Drug targeting to block specific functions of EBV-BILF1 has been suggested after engineering of the vGPCR to contain a binding site for a ‘tool’ compound (93). The conserved constitutive internalization among BILF1 receptors, suggests that a similar strategy could be applied in targeting EBV-infected cells and pigs infected with PLHV1 could serve as a model to test such a drug approach. EBV-BILF1 remains an orphan receptor lacking a defined ligand, but new technologies such as phage display technologies to identify novel ligands offer a potential means by which to target these receptors (93). Furthermore, using a target moiety fused to a toxic drug has shown potential in targeting vGPCRs (94). In the future, a development of a mini-pig model infected with PLHV1 may therefore provide the first suitable *in vivo* model to study the role of BILF1 in PTLD development and its potential use as drug target for the treatment of PTLD.

## Data Availability Statement

All sequencing data associated with this study are deposited at the European Nucleotide Archive under the project accession number: PRJEB42358.

## Ethics Statement

Ethical review and approval was not required for the animal study because all miniature swine experiments including diagnostic and euthanasia procedures were published previously in another study (DOI: 10.1182/blood.v97.5.1467) and were performed at an AAALAC International accredited institution in compliance with National Institute of Health guidelines for treatment of laboratory animals, and approved by the Institutional Animal Care and Use Committee. For the present study we only used the stored tissue material.

## Author Contributions

Conceptualization, KS, MMR, VK, BE, MV, JB, MJ. Data curation, MM, VK, KS, DPD, CH, EJJ, TP. Formal analysis, MM, KS, MMR, VK. Funding acquisition, VK, KS, MMR. Investigation, MM, KS, MMR, VK, DPD. Methodology, MM, VK, DPD, CH, JZ, EJJ, TP, MV, MJ, JB, KS. Project administration KS. Resources, MMR, VK, KS. Supervision, VK, KS, MMR. Validation, KS, MMR, VK. Visualization, MM. Investigation, MM, VK, DPD, CH, JZ, EJJ, KS. Writing—original draft preparation, MM, VK, MMR, KS. Writing—review and editing, MM, VK, CH, JZ, DPD, JB, MV, EJJ, TP, BE, MJ, MMR, KS. All authors have read and agreed to the submitted version of the manuscript.

## Funding

We acknowledge funding from the Slovenian Research Agency program P4-0053 and PhD funding for MM. This work was also supported by a Short-term scientific mission (STSM) grant from COST Action CA 18133 (ERNEST). Authors participate in the European COST Action CA 18133 (ERNEST). KS was funded by a postdoc grant from the Danish Council for Independent Research. TP and EJJ were funded by the P1-0207 Research Program grant, the J7-1818 Research Project grant to TP and the Z3-2650 postdoctoral fellowship to EJJ from the Slovenian Research Agency. MMR and KS were funded by The European Research council: VIREX (Grant agreement 682549, Call ERC-2105-CoG).

## Conflict of Interest

Author MJ was employed, in part, by the company the Vaccine Group, Ltd.

The remaining authors declare that the research was conducted in the absence of any commercial or financial relationships that could be construed as a potential conflict of interest.

## Publisher’s Note

All claims expressed in this article are solely those of the authors and do not necessarily represent those of their affiliated organizations, or those of the publisher, the editors and the reviewers. Any product that may be evaluated in this article, or claim that may be made by its manufacturer, is not guaranteed or endorsed by the publisher.
