# Supplementary material for: Epstein-Barr Virus-Encoded BILF1 Orthologues From Porcine Lymphotropic Herpesviruses Display Common Molecular Functionality
Source: Front Endocrinol (Lausanne). 2022 May 26;13:862940. doi: 10.3389/fendo.2022.862940 (PMC9204316; doi:10.3389/fendo.2022.862940)
Supplement: Supplementary file 1 [file DataSheet_1.docx]

Supplementary Material


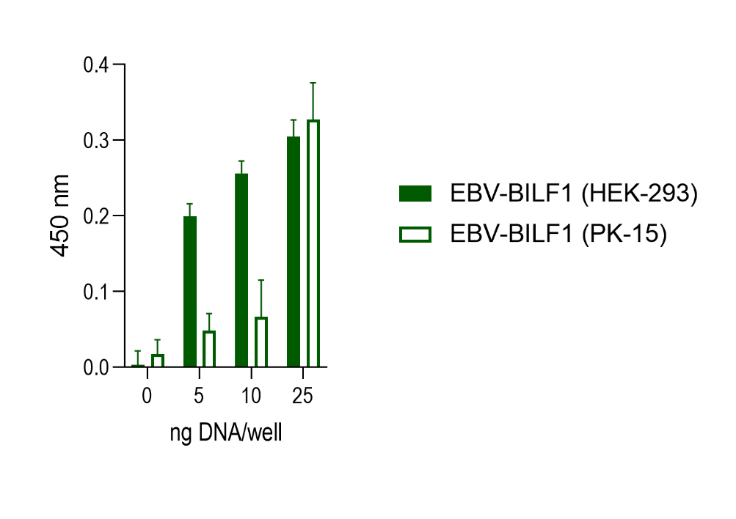


**Supplementary Figure 1.** **Comparison of receptor expression in HEK-293 cells and PK-15 cells.** Graph shows surface expression of EBV-BILF1 determined by cell-based ELISA using increasing concentrations of receptor DNA in HEK-293- (green bars) and PK-15 cells (white bars) (values are mean ± SEM).

**
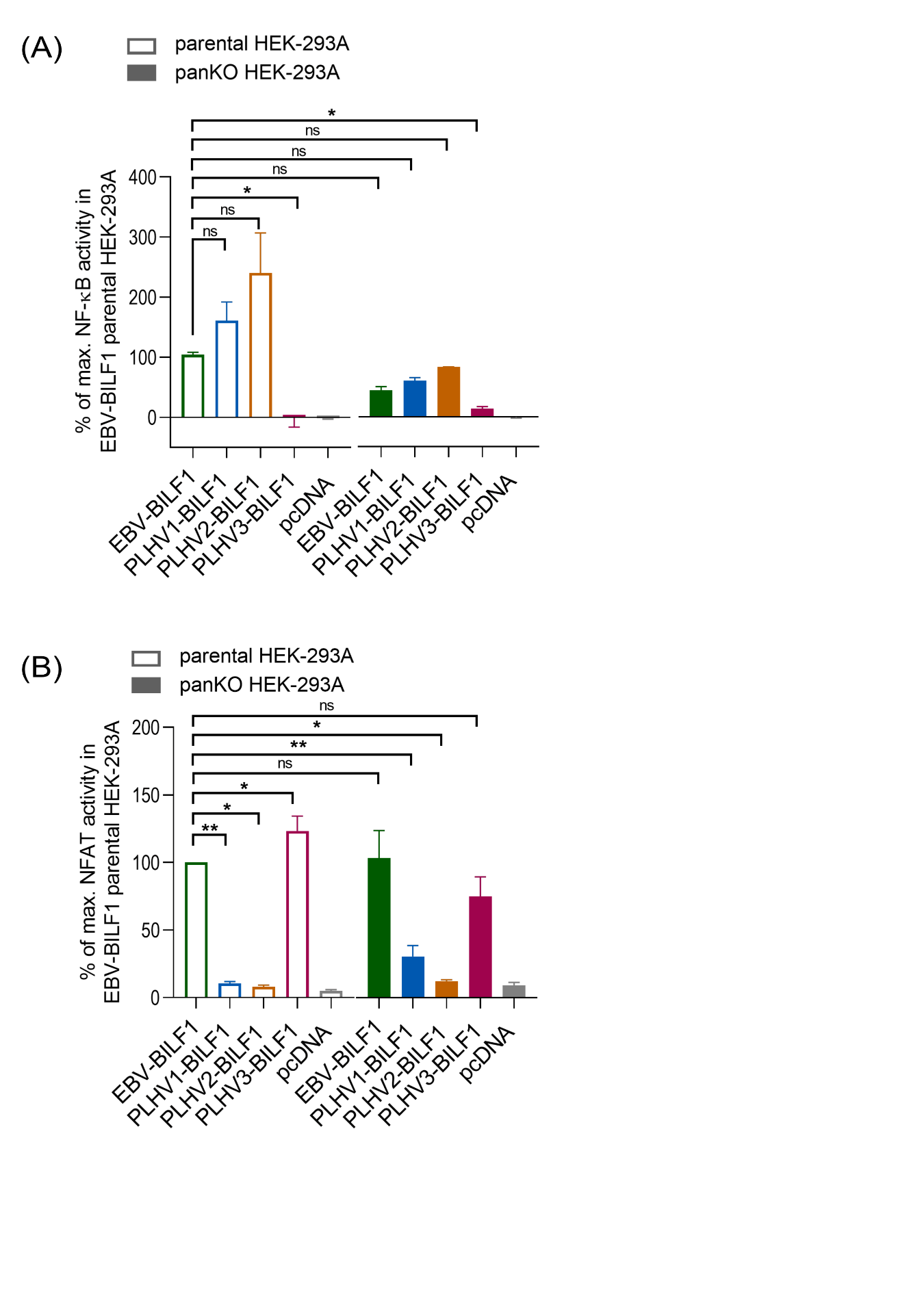
**

**Supplementary Figure 2. Maximal BILF1 receptor NF-κB and NFAT activity in CRISPR/Cas9 modified HEK-293A cells.** Bar diagrams represent the signaling of BILF1 receptors at the highest concentration (50ng/well). Graphs represent the receptor dependent (A) NF-κB and (B) NFAT activity relative to the maximal activity of EBV-BILF1 in parental cells (values are means ± SEM, n = 3). Statistical analysis was performed using unpaired Student t-test. **p*–value < 0.05; ***p*–value < 0.01; ns=non significant.


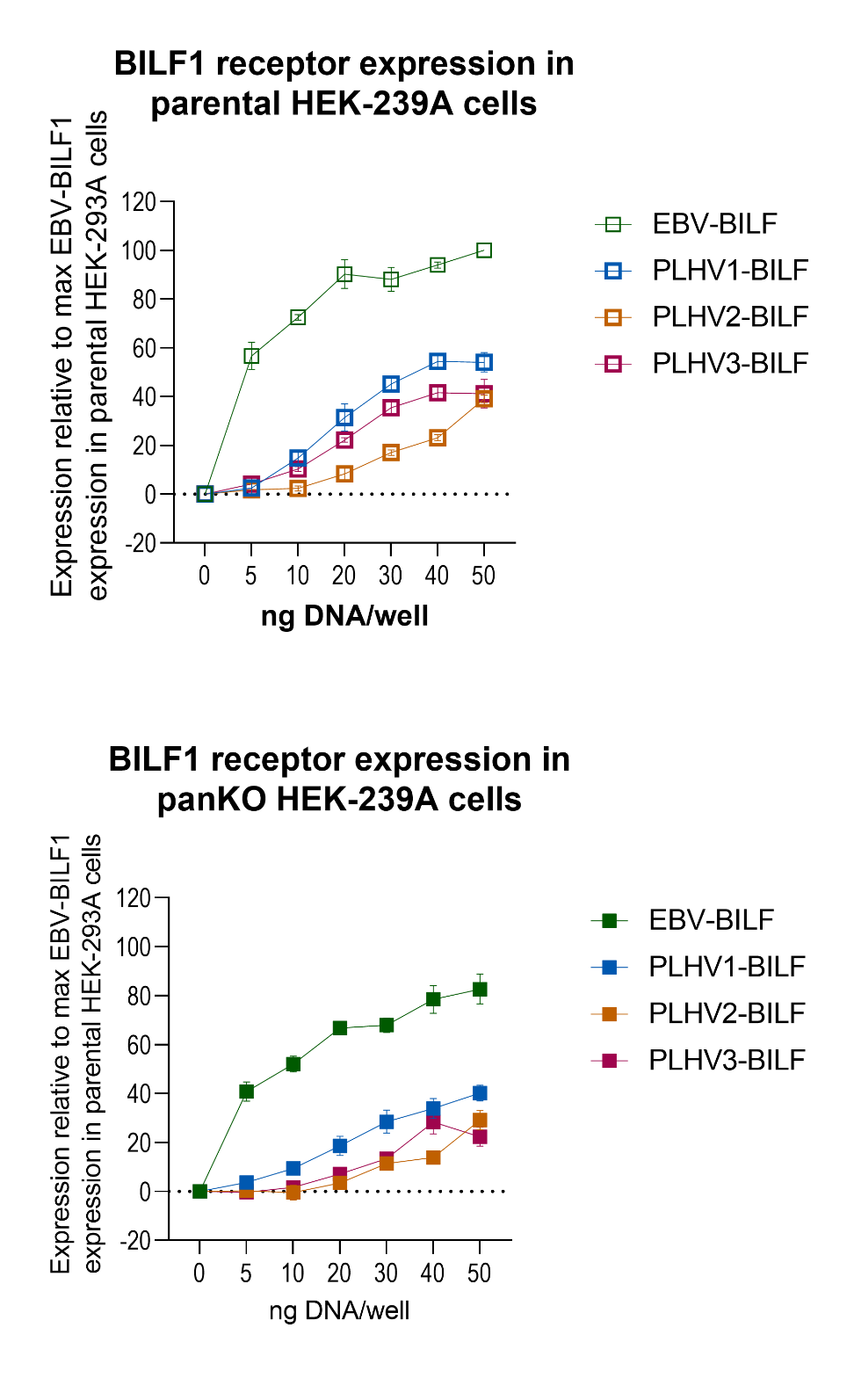


**Supplementary Figure 3.** **Receptor expression in parental HEK-293A and pan KO HEK-293A cells determined by ELISA in parallel with NFAT transcription factor assay.** Surface expression of PLHV BILF1 orthologues compared to EBV-BILF1 using cell-based ELISA at increasing concentrations of receptor DNA in parental HEK-293A and pan KO HEK-293A cells is shown. Cells were seeded and transfected in parallel with the cells used in NFAT transcription factor assay (values are mean ± SEM; *n* = 3).

**
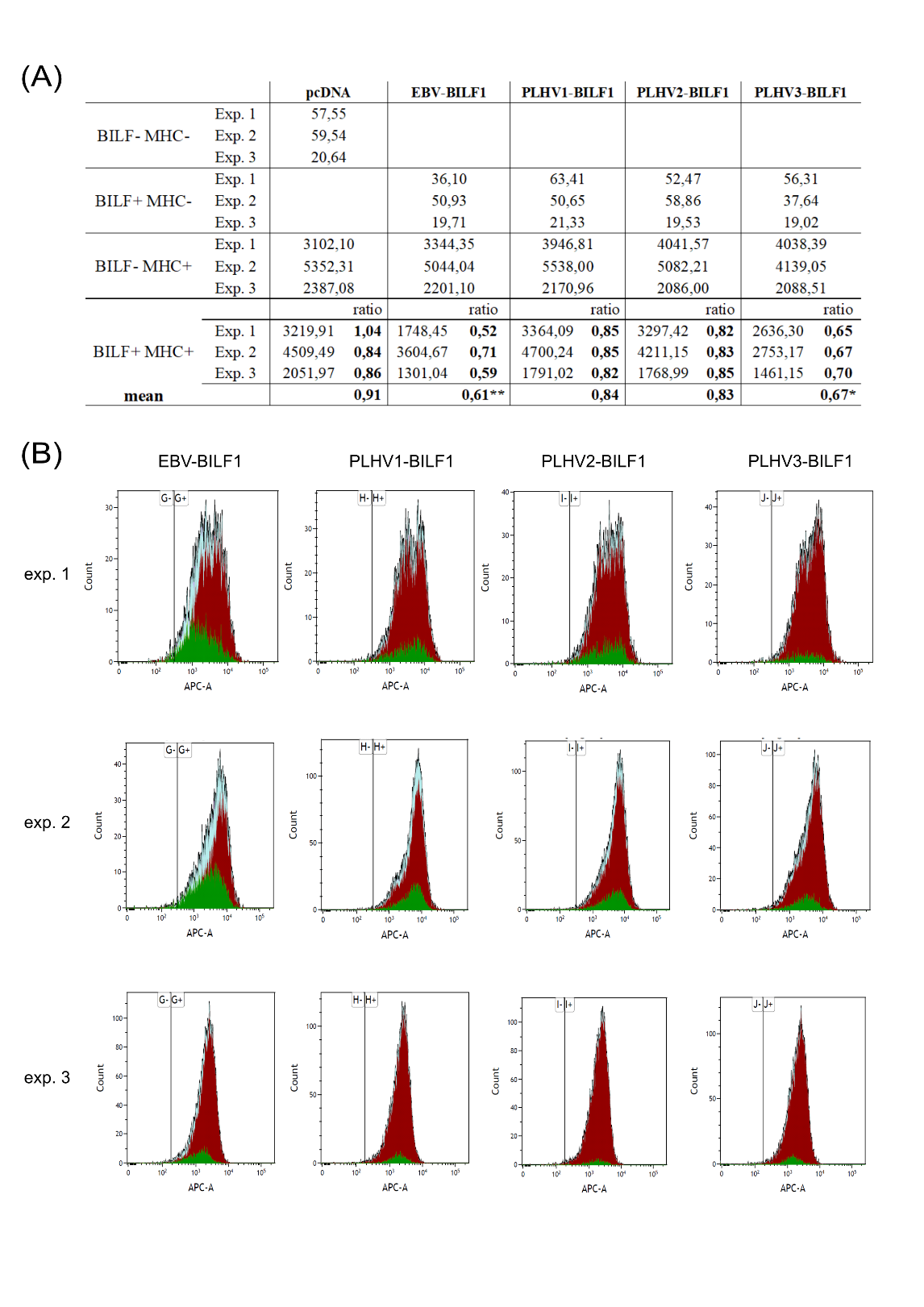
**

**Supplementary Figure 4. MHC-I downregulation in HEK-293 cells.** Using FACS analysis, we determined MHC-I downregulation in HEK-293 cells transiently transfected with BILF1 receptors. A) Table shows the MHC-I expression in different cell populations (BILF- and BILF+). Ratios represent the calculated value of MHC-I expression in BILF1 expressing cell / MHC-I expression in BILF1 not expressing cells. Statistical analysis was performed using one-way ANOVA analysis. **p*–value < 0.05; ***p*-value < 0.005. B) Representative histograms from three individual experiments. Two-color flow cytometry was used to analyze staining in the un-transfected FITC- population (red histogram) and transfected FITC+ population (green histogram)


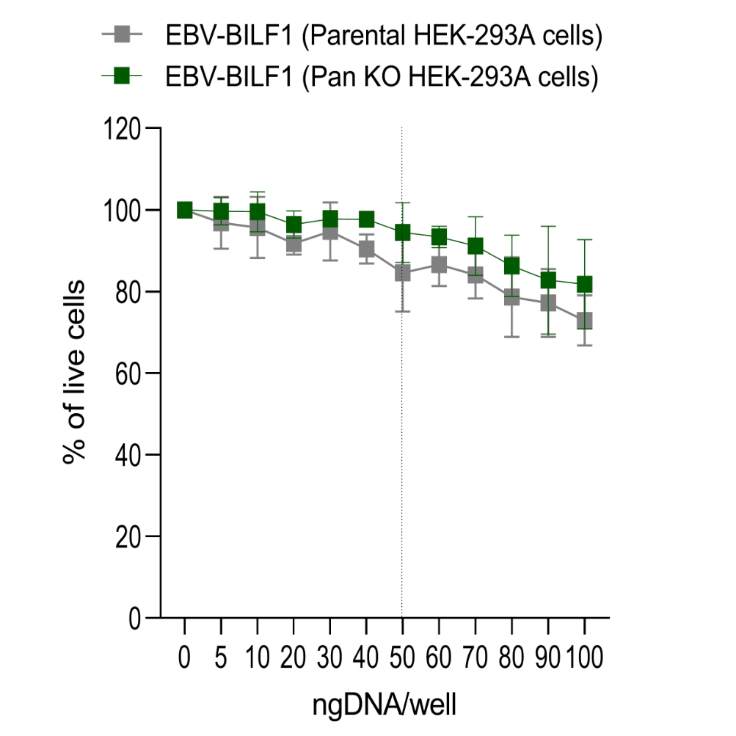


**Supplementary Figure 5.** **Viability assay of pan KO HEK-293A and parental HEK-293A cells.** Using Cell Titer Glo assay, we tested if the difference in parental HEK-293A and pan KO HEK-293 signaling is based on cell viability. Cells were transfected with increasing concentrations of EBV-BILF1 and 30ng/well of NF-kB transcription factor. In the actual transcription factor assay, we only used receptor concentrations up to 50ng DNA/well.


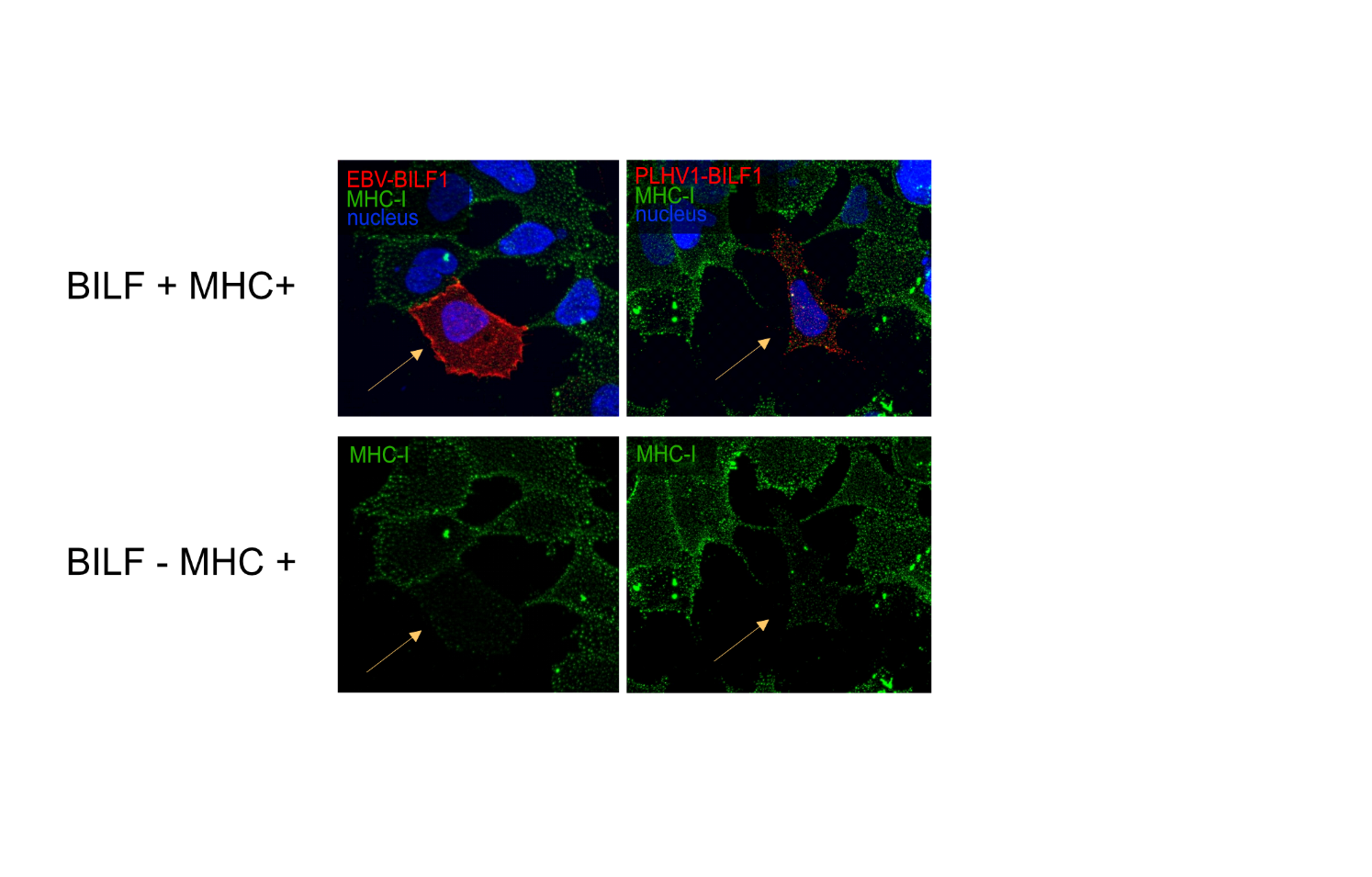


**Supplementary figure 6:** Two representative pictures used for the measurement of the MHC-I expression on transfected and non-transfected cells. MHC-I expression was measured using Image J.
